# Oral exposure to *Staphylococcus aureus* enterotoxin B could promote the Ovalbumin-induced food allergy by enhancing the activation of DCs and T cells

**DOI:** 10.3389/fimmu.2023.1250458

**Published:** 2023-10-16

**Authors:** Jin Yuan, Ping Tong, Xuanyi Meng, Yong Wu, Xin Li, Jinyan Gao, Hongbing Chen

**Affiliations:** ^1^ State Key Laboratory of Food Science and Resource, Nanchang University, Nanchang, China; ^2^ Sino-German Joint Research Institute, Nanchang University, Nanchang, China; ^3^ College of Food Science & Technology, Nanchang University, Nanchang, China

**Keywords:** Staphylococcus aureus enterotoxin B, food allergy, dendritic cells, T cells, gut microbiota

## Abstract

**Introduction:**

Recent work highlighted the importance of environmental contaminants in the development of allergic diseases.

**Methods:**

The intestinal mucosal barrier, Th (helper T) cells, DCs (dendritic cells), and intestinal flora were analyzed with flow cytometry, RNA-seq, and 16s sequencing in the present study to demonstrate whether the exposure of enterotoxins like *Staphylococcus aureus* enterotoxin B (SEB) in allergens could promote the development of food allergy.

**Results and discussion:**

We found that co-exposure to SEB and Ovalbumin (OVA) could impair the intestinal barrier, imbalance the intestinal Th immune, and cause the decline of intestinal flora diversity in OVA-sensitized mice. Moreover, with the co-stimulation of SEB, the transport of OVA was enhanced in the Caco-2 cell monolayer, the uptake and presentation of OVA were promoted in the bone marrow dendritic cells (BMDCs), and Th cell differentiation was also enhanced. In summary, co-exposure to SEB in allergens should be considered a food allergy risk factor.

## 1 Introduction

The prevalence of food allergy has been increasing in the last decades. It represents a significant threat to patients’ health and carries a substantial medical burden (1). The causes of food allergy are complex and result from the interaction between genetic and environmental factors. Exposure to bacterial toxins was now considered one of the risks of food allergy (2, 3). These bacteria or their toxins could impair the mucosal barrier and local immune balance like the skin, intestine, and nasal cavity, thereby affecting the recognition and uptake of allergens and finally leading to immune intolerance of food allergens (4, 5). Accordingly, there is an urgent need to indicate the potential correlation between bacterial toxins and food allergies.

*Staphylococcus aureus* (*S. aureus*) is one of the most prevalent bacterial pathogens in food-borne diseases, second only to *Salmonella* in the incidence of food contamination (6). Contaminations of *Staphylococcus aureus* enterotoxin B (SEB) can be easily found in moist foods consisting of starch and protein, including dairy, pork, beef, lamb, poultry, and egg. SEB could be brought in directly by infected animals or poor hygiene during production, retail, and storage (7). It could cause acute vomiting, dizziness, and diarrhea, and in more cases, may lead to some potentially asymptomatic infections. Since various allergen-containing foods can be contaminated with SEB, the association between SEB and food allergies needs to be examined.

The essential role of SEB in allergic diseases, including asthma, rhinitis, and atopic dermatitis, has been proven and characterized by more epidemiological evidence (2, 8, 9). Several immunological pathways activated by SEB could influence the development of allergy. The crosstalk between dendritic cells (DCs) and oligoclonal T cells could be active by SEB unspecifically, leading to the rapid differentiation of helper T (Th) cells and the release of cytokines (10) through the interaction of Major Histocompatibility Complex II (MHC-II) and T cell receptor (TCR) molecules (11, 12). The injury of the intestinal barrier (13) and a decrease in the abundance and microecology of gut microbiota (14) induced by SEB could also be an approach for allergens to intrude into the internal environment. The intestinal epithelial barrier dysfunction caused by bacterial toxins like cholera toxin and SEB (15, 16) with increased permeability to allergens offers the possibility of the initiation of food allergy (17). Therefore, we hypothesized that co-exposure to SEB and allergens might promote the development of food allergies through intestinal barrier damage, intestinal microbiota dysregulation, and DC-T cell abnormal activation.

To complement and refine this evidence and hypothesis, the potential damage to the intestinal and disturbs of mucosal immunity induced by co-exposure to SEB and allergens need to be further evaluated to define its role in developing food allergies. Hence, the influence on the intestinal mucosal barrier, intestinal microbiota, and DC-T cell communication induced by the co-exposure to OVA and SEB *in vivo and in vitro* were investigated in this study. It could provide new evidence on whether the intestinal barrier’s damage, microbiota dysfunction, and the activation of DCs and T cells were induced by bacterial toxins that play a vital role in the development of food allergy.

## 2 Materials and methods

### 2.1 Mice

The 5- to 6-week-old female BALB/C mice were purchased from Hunan SJA Laboratory Animal Co., Ltd, maintained in a specific pathogen-free environment, and had free access to food and water. All animal experiments and handling procedures were approved by the Animal Care and Use Committees of Nanchang University and were performed in accordance with institutional guidelines (Animal Ethics Code: 2021-0308-035).

### 2.2 Materials

The OVA was purified from hen eggs using an anion exchange column (18). The SEB was purchased from Toxin Technology, Inc., Florida, USA (Catalog # BT202). Lipopolysaccharide (LPS) was purchased from Thermo Fisher Scientific.

### 2.3 Food allergy mouse model

**Figure 1:**
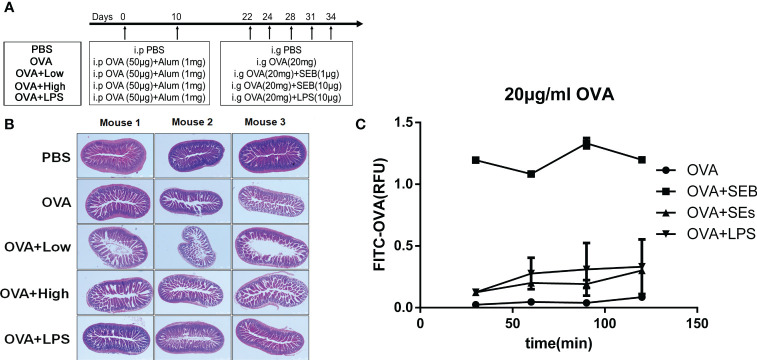
Intestinal injury was aggravated by exposure to SEB in sensitized mice. **(A)** The procedure of animal experiment. **(B)** The 5 to 6-week-old female BALB/C mice were received twice peritoneal injections with alum (1 mg) and OVA (50 μg) on day 0 and day 10 and challenged five times through oral gavage from day 22 to day 34 with PBS (PBS group), OVA (20 mg) only (OVA group), OVA (20 mg) added with SEB (1 μg) (OVA+Low group), OVA (20 mg) added with SEB (10 μg) (OVA+High group) or OVA (20 mg) added with LPS (10 μg) (OVA+LPS group). The jejunum tissues were collected and stained with HE. **(C)** The transportation of FITC-conjoint-OVA (20 μg/ml) through a Caco-2 cell monolayer model. Data are presented as mean ± SEM.

The procedure of the animal experiment is shown in **Figure 1A**. The mice were sensitized with twice peritoneal injections with alum (1 mg) and OVA (50 μg) on day 0 and day 10 and challenged five times from day 22 to day 34 with 20 mg OVA (OVA group), 20 mg OVA added with 1 μg SEB (OVA+Low), 20 mg OVA added with 10 μg SEB (OVA+High), or 20 mg OVA added with 10 μg LPS (OVA+LPS). After the last challenge, the jejunum and colonic contents were collected for RT-PCR and microbial genome analysis. The mice sensitized and challenged with PBS (PBS group) were used as a negative control. This model was repeated twice, with six mice in each group.

### 2.4 The Hematoxylin-eosin staining of jejunum tissue in mice

After the last challenge (Day 34), the jejunum tissues from mice were collected embedded in paraffin and cut into 5 μm sections. The sections were then deparaffinized in xylene, ethanol, and distilled water. The sections were soaked in hematoxylin dye (0.2% hematoxylin in water) for 5 min. After washing, the sections were washed in ethanol containing 1% hydrochloric acid for seconds and in eosin solution (0.5% eosin in water) for 1 min.

### 2.5 RT-PCR

Total RNA was purified from jejunum tissues in mice with TRIzol (purchased from Thermo Fisher Scientific) and then reverse transcribed with a PrimeScript™ RT reagent Kit (purchased from TakaRa). The procedure was performed following the Kits of TRIzol and RT reagent. Briefly, to isolate the total RNA, the jejunum was thoroughly homogenized in 1 ml TRIzol for 5 minutes, added with 0.2 mL of chloroform, and then centrifuged to remove the lower red phenol chloroform. Add 0.5 mL of isopropanol to the aqueous phase and discard the supernatant with a micropipette after centrifuge. The total RNA was diluted and incubated with a gDNA Eraser for 5 min before reverse transcription through an RT Mix containing RT Enzyme and Primer.

For the evaluation of the tight-junction proteins and differentiation of Th cells in the jejunum, the mRNA transcripts for *ZO-1*, *Occludin*, *Claudin-2*, *IFN-γ*, *IL-13*, *TGF-β*, *TRIF-4*, and *Gata3* were quantified by real-time PCR using QuantiNova SYBR Green PCR (purchased from QIAGEN) in the QuantStudio 3 system (Thermo Fisher Scientific) as the manufacturer’s instructions. Real-time cycler conditions were followed with the handbook from QuantiNova SYBR Green PCR. The data was calculated by using the 2−ΔΔCT method, and the primer sequences were displayed in **Supplementary Table 1**. The difference in the target gene expression was corrected to obtain the relative expression level ( $\Delta \Delta CT_{Sample}$ ), after the CT values of the target gene and the housekeeping gene (*Gapdh*) in samples were obtained. The fold changes of target genes in each group were involved in statistical comparison.


$$\Delta CT_{Sample}=CT_{Taget}-CT_{Gapdh}$$

$$\Delta \Delta CT_{Sample}=\Delta CT_{Sample}-\Delta CT_{Control}$$

$$Fold\;Change=2^{-\Delta \Delta CT}$$

### 2.6 Caco-2 cell monolayer model

The Caco-2 cell monolayer was established using Transwell plates (purchased from NEST Biotechnology Co.LTD) through a 21-day culture in complete DMEM medium (Dulbecco’s modified eagle medium, purchased from CellMax, added with 10% fetal bovine serum). After a 21-day culture, the medium was replaced with Hank’s solution (purchased from Beijing Solarbio Science & Technology Co., Ltd), and the transmembrane resistance value of the Caco-2 cell monolayer was tested until stable. The FITC-OVA (20μg/ml) was added to the upper compartment of the Transwell chamber together with SEB (50 ng/ml), SEs (supernatant of 10^6^/ml *S. aureus*), or LPS (1 μg/ml) (19). The FITC-OVA in the lower compartment was tested through a fluorophotometer after being cultured for 30 min, 60 min, 90 min, and 120 min. FITC-OVA was prepared using a FITC conjugation kit (purchased from Sigma). Briefly, OVA was incubated with FITC dye for 2 h in a sodium bicarbonate solution. The FITC-conjunct OVA was isolated through a Sephadex G-25M column.

### 2.7 BMDCs culture and isolation of small intestinal epithelial cells and spleen naïve T cells

Bone marrow dendritic cells (BMDCs) were generated from mice’s bone marrow (BM) cells (20, 21). Briefly, BM cells were flushed out from the femurs and tibias using RPMI 1640 (Roswell Park Memorial Institute, medium, purchased from CellMax). BM cells were cultured in a complete culture medium (RPMI 1640 supplemented with 10% FBS) containing 20 ng/ml GM-CSF (purchased from Peprotech) and 10 ng/ml IL-4 (purchased from Peprotech) for 1 week. The spleen naïve Th cells were enriched by Naïve CD4^+^ T Cell Isolation Kit (purchased from Miltenyi Biotec) after being ground through a cell strainer (70 μm) from mice. The purity of enriched naïve Th cells is shown in **Figure S1A**. The small intestinal epithelial cells were collected from mice by dissolving in Hank’s solution for 30 minutes, then enriched using CD326 Isolation Kit (purchased from Miltenyi Biotec) (22). The small intestine was washed in PBS and cut into 5 cm pieces. These intestinal pieces were added in Hank’s solution containing 5mM EDTA (purchased from Beijing Solarbio Science & Technology Co., Ltd) and 1mM DTT (purchased from Beijing Solarbio Science & Technology Co., Ltd) and shaken for 30 min in 37°C, 250 rpm. Pieces were removed using 70 μm cell strainers. The epithelium was collected after trypsin digestion for 5 min and incubated with anti-CD326 microbeads for 15 min. The CD326^+^ epithelial cells were separated with a magnetic column according to the CD326 Isolation Kit.

### 2.8 Cell stimulation

The collected BMDCs were co-cultured with enriched small intestinal epithelial cells. They were then stimulated with PBS, OVA (50 μg/ml), OVA (50 μg/ml) +LPS (1μg/ml), OVA (50 μg/ml) +SEB (50ng/ml), or OVA (50 μg/ml) +SEs (supernatant of 10^6^/ml *S. aureus*) for 8 h, respectively. And then, they were collected for flow cytometry.

The enriched naïve T cells were co-cultured with BMDCs pre-stimulated with OVA (50 μg/ml) for 8 h under the stimulation with PBS, OVA (50 μg/ml), SEB (50 g/ml), or OVA+SEB for 24 h. Afterward, the expression of T-bet, GATA3, and Foxp3 in CD4^+^ T cells was evaluated by flow cytometry.

### 2.9 FACS and antibody

The stimulated BMDCs and naïve T cells were then used in flow cytometry analysis with CytoFLEX Flow Cytometer (Beckman Coulter) and rendered using flowJo software (FlowJo V10.6.2, Becton Dickinson & Company). The following anti-murine antibodies were used for flow cytometry: PE-CD11c (N418), FITC-I-A/I-E (M5/114.15.2), PE -CD103 (2E7), PerCP-Py5.5-CD86 (PO3), APC-CD40 (3/23) and BV605-CD4 (GK1.5) (all purchased from Biolegend); AF647-T-bet (04–46), BV421-GATA3 (L50-823) and FITC-Foxp3 (PC61) (purchased from eBioscience, Thermo Fisher Scientific). The Fixation and Permeabilization Solution (purchased from BD) was used to assess intracellular markers.

### 2.10 RNA sequencing and data analysis

The total RNA from BMDCs was extracted after BMDCs were sorted through flow cytometry (CytoFLEX SRT, Beckman Coulter) and stimulated with PBS, OVA (50 μg/ml), or OVA (50 μg/ml) +SEB (50ng/ml). And the mRNA containing Poly A underwent random N6 primer reverse transcription after being enriched by Oligo dT Selection. The double-stranded cDNA was amplified and sequenced on the DNBSEQ™ platform after ER/A-tailing and bubble adapter ligation. The quality control is then performed on the raw reads to determine whether the sequencing data is suitable for subsequent analysis. The gene quantification analysis and other investigations based on gene expression (principal component, correlation, differential gene screening), significant enrichment analysis on differentially expressed genes among the screened samples, significance enrichment analysis of pathway, clustering, protein interaction networks, and transcription factors were performed using Dr.Tom online system of BGI Genomics (https://biosys.bgi.com, could be turned into English) after the filtered clean reads were aligned to the reference sequence.

### 2.11 16S rRNA gene sequencing and data analysis

The 16S rRNA in the microbial genome of the colonic contents from mice was obtained and retro-transcribed to 16S rDNA. Then, the amplified 16S rDNA was sequenced through the HiSeq system after it was purified by Agencourt AMPure XP microbeads. The clean data were then analyzed based on OTU (Optical Transform Unit) clustering and KEGG (Kyoto Encyclopedia of Genes and Genomes) database resource through the online microbe system of BGI Genomics (http://meta.bgi.com/microbe, which could be turned into English) after quality-filtered.

### 2.12 Statistical analysis

All data were analyzed using GraphPad Prism 6 (GraphPad Software, Inc, Dotmatics). Statistical significance was determined with a one-way ANOVA (Analysis of Variance) analysis of variance with Tukey’s multiple comparisons test (*p<.05, **p<.01, ***p<.001, ****p<.0001).

## 3 Results

### 3.1 The intestinal injury could be enhanced by co-exposure to SEB in sensitized mice

The intestinal barrier injury in jejunum tissues induced by SEB and OVA-allergic mice was evaluated by histopathological analysis using HE staining. In **Figure 1B**, the jejunum from the PBS group exhibited normal morphology, intestinal villi, and well-arranged epithelial cells. The intestinal barrier showed some disruption of the intestinal villi in the OVA group after the challenge with the allergen. More destruction of the intestinal epithelium, loss of goblet cells, sporadic loss of nuclei staining, villous blunting, distortion of the crypt, and overall tissue architecture could be observed in the small intestines of OVA+Low and OVA+High groups compared to the OVA groups. The Caco-2 cell monolayer was established to further evaluate the transportation of FITC-OVA through the intestinal epithelial barrier. The transportation of FITC-OVA was significantly enhanced by the co-stimulation of SEB compared to that in OVA groups (**Figure 1C**). The co-stimulation of LPS and SEs had no significant difference compared to the OVA group.

### 3.2 Co-exposure to SEB induced more tight-junction proteins impairs and Th1 differentiation

**Figure 2:**
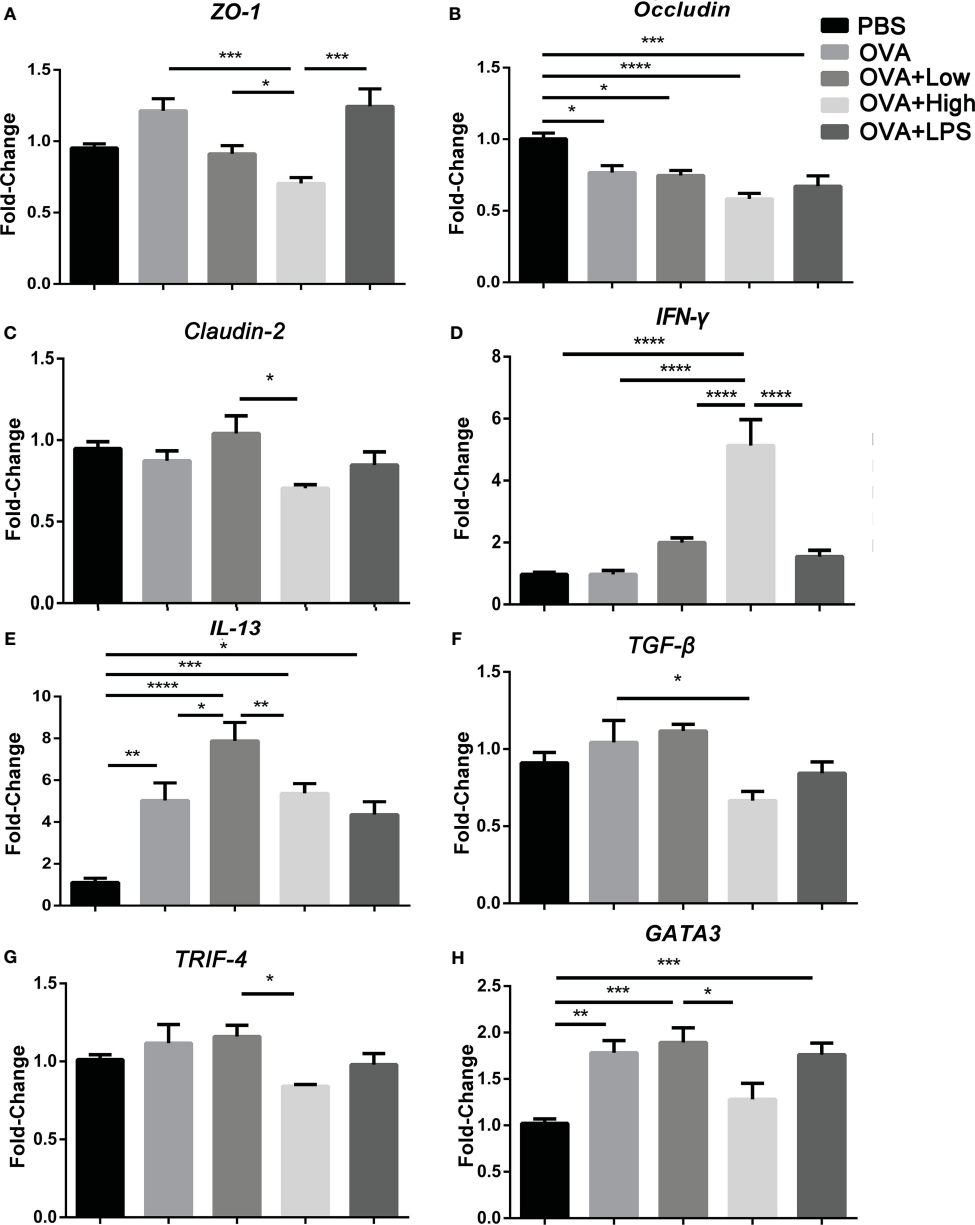
The mRNA transcripts for ZO-1 **(A)**, Occludin **(B)**, Claudin-2 **(C)**, IFN-γ **(D)**, IL-13 **(E)**, TGF-β **(F)**, TRIF-4 **(G)**, and Gata3 **(H)** were quantified by real-time PCR. Data are presented as mean ± SEM; statistical significance was determined with one-way ANOVA analysis of variance with Tukey’s multiple comparisons test (*p<.05, **p<.01, ***p<.001, ****p<.0001).

As more intestinal barrier damage existed in the SEB groups, the jejunum from mice was further collected for RT-PCR (**Figure 2**). More expression of *IL-13* or *GATA3* and less expression of *occludin* was observed in the jejunum of the OVA group compared to PBS group (**Figures 2B, E, H**). And the gene expression of *IL-13* and *IFN-γ* was significantly increased in the OVA+Low group compared to the OVA group (**Figures 2D, E**). Less occluding, ZO-1, and TGF-β gene expression were found in the OVA+High group compared to the OVA group (**Figures 2A–C**). The expression of *IFN-γ* was significantly increased in the OVA+High group compared to the OVA groups (**Figure 2D**). Compared to the OVA+Low group, the mice in the OVA+High group expressed more *IFN-γ* and less *TRIF*, *IL-13*, *GATA3*, and *TGF-β* in the jejunum. The OVA+Low group showed no significant change compared to the OVA group in **Figure 2**.

### 3.3 The exposure to SEB partly decreased the abundance of caecal microflora in food-allergy mice

**Figure 3:**
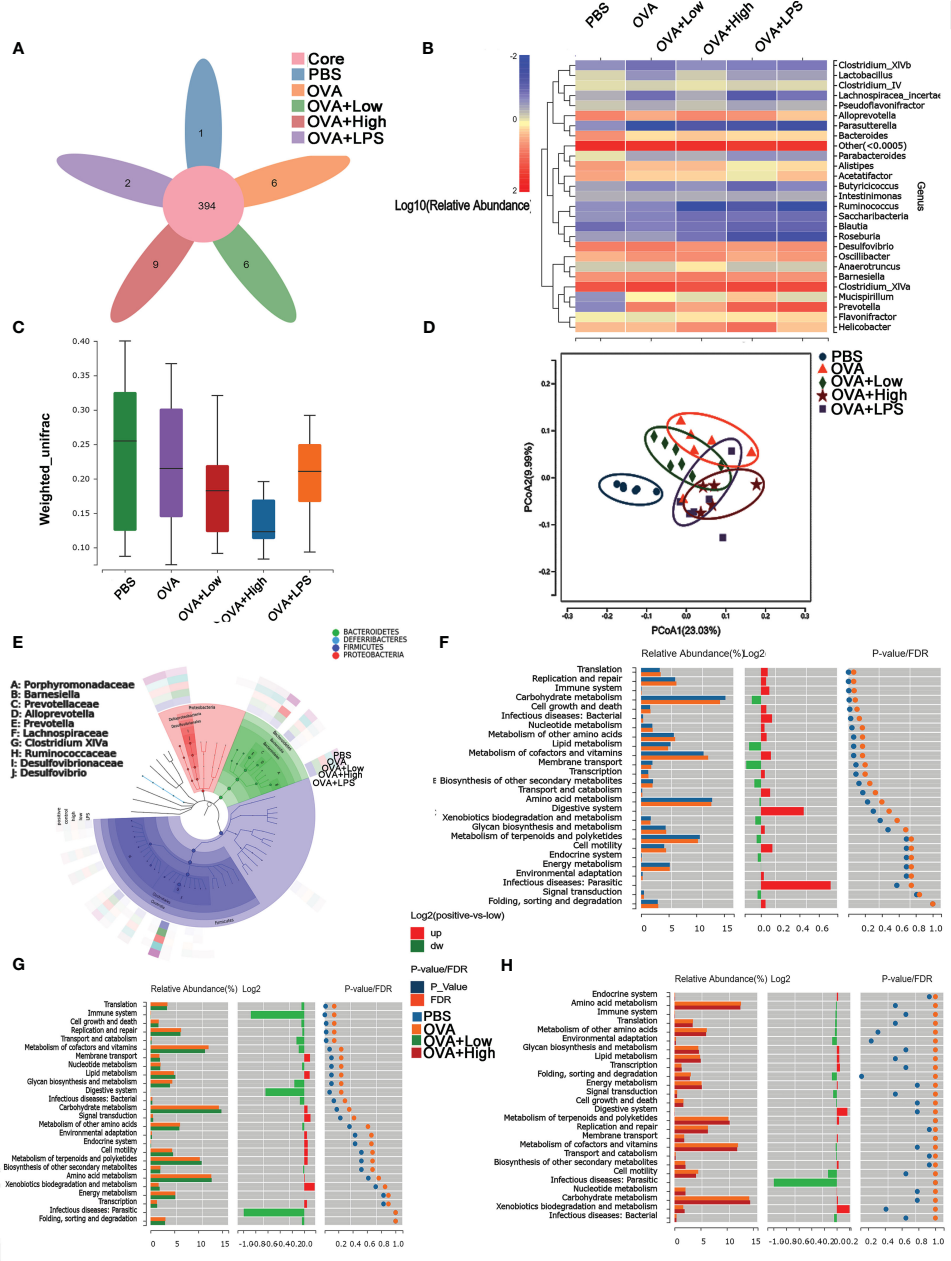
The diversity of caecal microflora in mice was performed using massively parallel pyrosequencing of 16S rDNA amplicons. **(A)** The Venn Diagram shows the numbers of different species in each group. **(B)** The Cluster analysis of characteristic genes. **(C)** Shannon’s Diversity Index assessed the diversity indices. **(D)** The PCoA (Principal Co-ordinates Analysis) was performed according to Bray−Curtis dissimilarity. **(E)** LEfSe analysis of caecum microflora. **(F–H)** The differences in functional caecal microflora between groups were performed based on KEGG.

The community structure analysis of caecal microflora was performed by using massively parallel sequencing of 16S rDNA amplicons (**Figure 3**). In PBS, OVA, OVA+Low, OVA+High, and OVA+LPS groups, 1, 6, 6, 9 and 2 gut bacteria were unique compare to each other, respectively (**Figure 3A**). **Figure 3B**, **E** show the heat map of species composition at the species level among other intestinal bacterial groups in each group. The results showed that compared with the PBS group, *Bacteroides sartorii*, *Alistipes onderdonkii*, *Acetatifactor muris*, *Parabacteroides merdae*, *Clostridium colinum*, *Bacteroides vulgatus*, and *Parasutterella excrementihominis* were significantly decreased in OVA group. Compared to the OVA group, *Parasutterella excrementihominis* was further reduced in the OVA+Low and OVA+High groups. Meanwhile, *Prevotella dentalis*, *Mucispirillum scheduler*, and *Alistipes finegoldii* were more abundant in the OVA group compared to the PBS group. *Prevotella dentalis* increased in the OVA+Low and OVA+High groups compared with the OVA group. The results of intestinal microbiota richness based on Beta diversity analysis showed that SEB co-exposure could reduce microbiota diversity compared with the OVA group (**Figure 3C**). The PCoA (Principal Co-ordinates Analysis) was performed according to Bray−Curtis dissimilarity and calculated based on the abundance of OTUs, which showed the co-exposure to SEB and LPS could partly reverse the disparities between the intestinal microbiota of the OVA group and PBS group (**Figure 3D**). Analysis of differences in functional caecal microflora between groups was performed based on KEGG, to find out which kinds of functional microorganisms were regulated under the stimulation of SEB (**Figures 3F-H**). Relative abundance of intestinal flora with functions in translation, replication and repair, immune system, cell growth, and infectious disease was significantly upregulated in the OVA group compared to the PBS group (**Figure 3F**). The co-exposure of a high dose of SEB (OVA+High group) led to no significant changes in functional intestinal flora when compared to the OVA group (**Figure 3G**). However, the co-exposure of a low dose of SEB (OVA+Low group) appeared to partially reverse the changes in functional microbiota in the OVA group (**Figure 3H**).

### 3.4 The stimulation of SEB promoted the uptake of OVA in BMDCs and the activation of BMDCs

**Figure 4:**
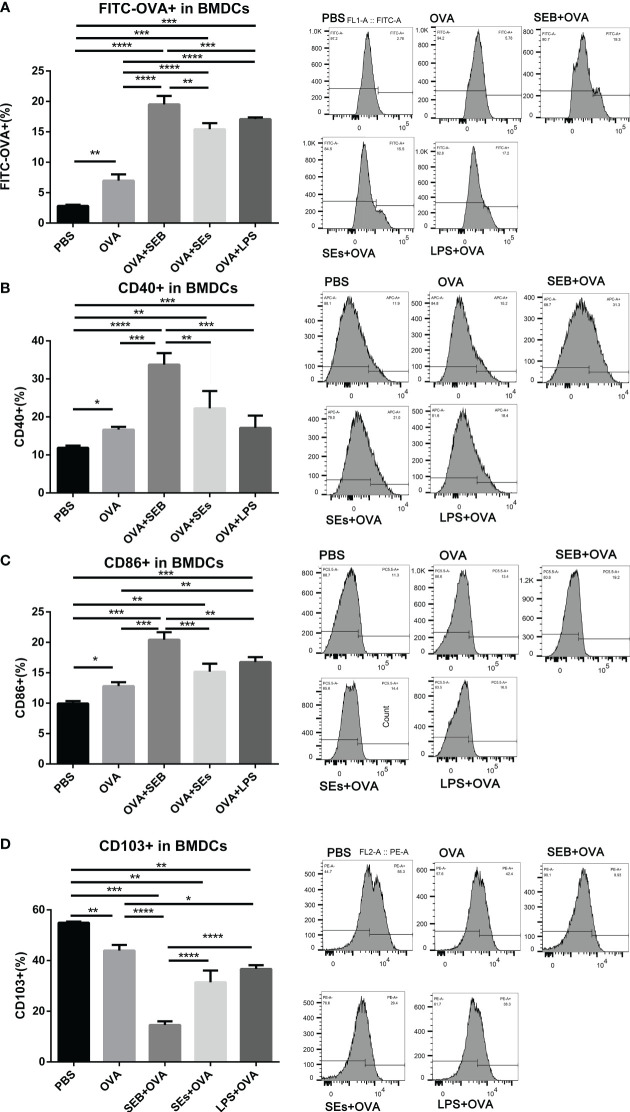
The stimulation of SEB promoted the uptake of OVA in BMDCs. The BMDCs were co-cultured with isolated small intestinal epithelial cells. Then they were stimulated with PBS, OVA (50 μg/ml), OVA (50 μg/ml) +LPS (1μng/ml), OVA (50 μg/ml) +SEB (50ng/ml), or OVA (50 μg/ml) +Sas (supernatant of 106/ml S. aureus) for 8 h, followed by the analysis of the contents of FITC-OVA **(A)** and expression of CD40 **(B)**, CD86 **(C)** and CD103 **(D)** using flow cytometry. Data were presented as mean ± SEM; statistical significance was determined with one-way ANOVA analysis of variance with Tukey’s multiple comparisons test (*p<.05, **p<.01, ***p<.001, ****p<.0001).

The uptake of FITC-OVA and expression of surface markers in BMDCs co-cultured with enriched epithelial cells was evaluated by flow cytometry (**Figure 4**). The uptake of FITC-OVA in BMDCs was significantly increased under the stimulation of OVA added with SEB, SEs, and LPS, compared to OVA group (**Figure 4A**). Moreover, the expression of CD40 and CD86 in BMDCs was significantly increased, and CD103 was decreased considerably under the stimulation of OVA compared to the PBS group (**Figures 4B–D**). The stimulation of SEB, SEs, and LPS significantly reduced the expression of CD103 in BMDCs compared to the OVA group (**Figure 4D**). Under the co-stimulation of SEB and OVA, the uptake of FITC-OVA and expression of CD40 and CD86 was significantly increased, and the expression of CD103 was significantly decreased compared to the OVA group. The expression of CD40 and DC86 was significantly increased, and the expression of CD103 was significantly reduced in the SEB+OVA group compared to other groups.

### 3.5 The co-stimulation of SEB and OVA promoted the differentiation of Th1 and Th2 cells

**Figure 5:**
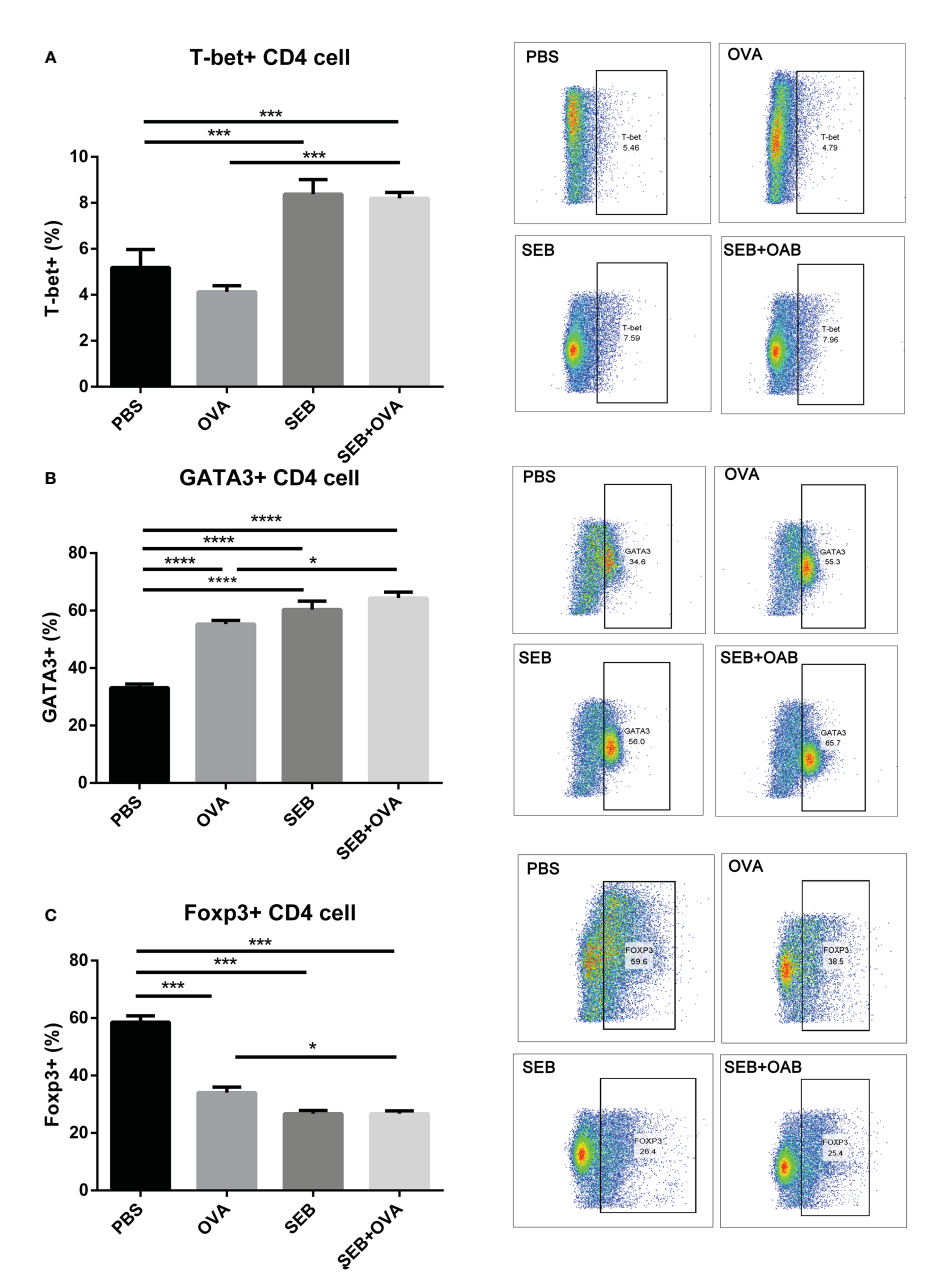
The stimulation of SEB promoted activation of Th cells. The enriched naïve T cells were co-cultured with BMDCs pre-stimulation with PBS, OVA (50 μg/ml), SEB (50 ng/ml), and OVA+SEB for 24 h, respectively. And the expression of T-bet, GATA3, and Foxp3 in CD4^+^ T cells was evaluated. Data were presented as mean ± SEM; statistical significance was determined with one-way ANOVA analysis of variance with Tukey’s multiple comparisons test (*p<.05, ***p<.001, ****p<.0001).

To further investigate the promotion effect on BMDCs of co-stimulation of SEB, a co-cultural model of BMDCs-naïve Th cell was established (**Figure 5**). The stimulation of OVA could significantly increase the expression of GATA3 and decreased the expression of Foxp3 in Th cells compared to the PBS group (**Figures 5B, C**). The gating strategy of Th cells was shown in **Figure S1B**. Significant increases in frequencies of T-bet^+^ and GATA3^+^ Th cells were observed under co-stimulation of OVA and SEB compared to the OVA group (**Figures 5A, B**). And the expression of Foxp3 in Th cells was significantly decreased under the stimulation of OVA and SEB compared to the OVA-only group (**Figure 5C**). There was no significant difference between the SEB+OVA group and the SEB group.

### 3.6 The stimulation of SEB upregulates gene sets associated with immune responses to OVA stimulation in BMDCs

**Figure 6:**
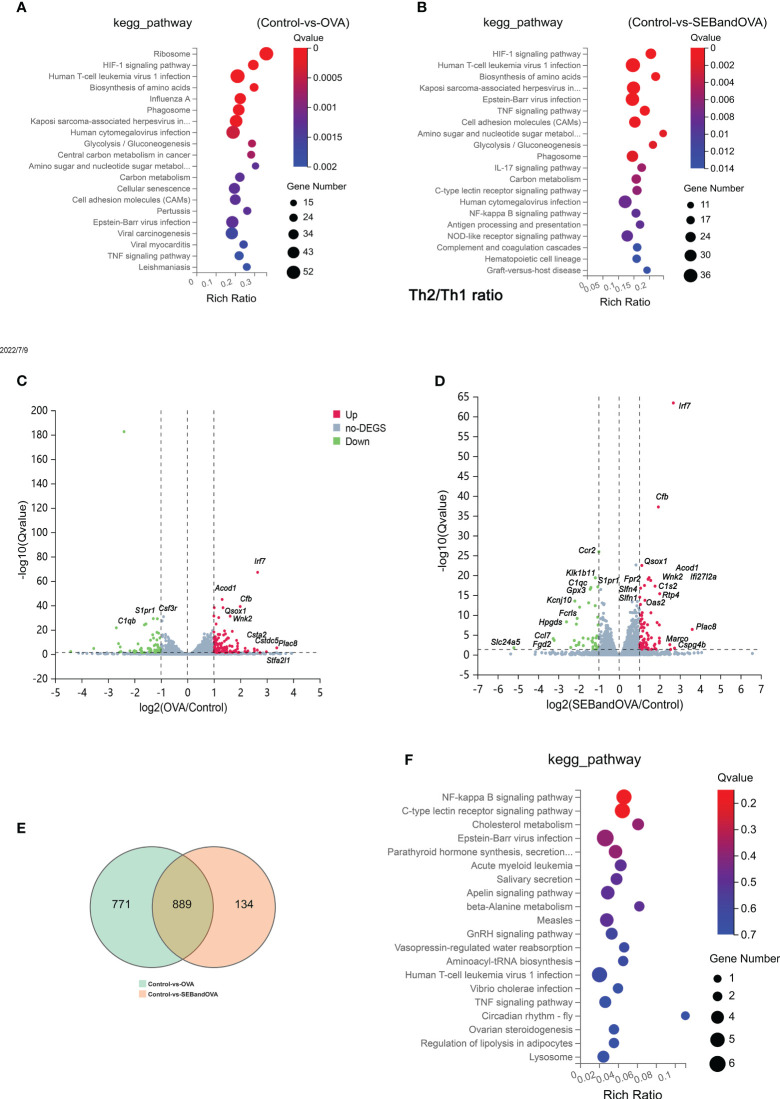
The stimulation of SEB upregulates gene sets associated with immune responses to OVA stimulation in BMDCs. The total RNA from BMDCs stored through flow cytometry and stimulated with PBS, OVA (50 μg/ml), or OVA (50 μg/ml) +SEB (50ng/ml) undergo RNA Sequencing analysis. **(A, B)** The enriched KEGG pathways between Control and OVA or Control and OVA+SEB were identified. **(C, D)** Volcano Plot showing log fold-change and average gene expression between Control and OVA or Control and OVA+SEB. **(E)** The Venn Diagram of different genes was between Control VS OVA and Control VS SEBandOVA. **(F)** The expression of 134 genes was regulated uniquely in the OVA+SEB group and was identified in the KEGG pathway.

To clarify the potential mechanisms that BMDCs tend to present more allergens to T cells and promote Th polarization under the stimulation of SEB, the stored mouse BMDCs stimulated with OVA or OVA+SEB were collected and undergo RNA-seq analysis (**Figure 6**). To investigate biological themes among our list of differentially expressed genes, we used the KEGG database resource to identify different pathways between Control and OVA (**Figure 6A**) or OVA and OVA+SEB (**Figure 6B**). It was found that regulated genes were intensely involved in signal transduction and the immune system both in stimulation with OVA or OVA+SEB. And in the Volcano Plot, log fold-change and average gene expression in log count percent were upregulated in stimulation with OVA or OVA+SEB (**Figures 6C, D**). The expression of 134 genes was regulated uniquely in the OVA+SEB group, mainly focused on the NF-κB pathway, C-type receptor pathway, and cholesterol metabolism (**Figures 6E, F**).

## 4 Discussion

The possible damage to the intestinal and abnormal activation of mucosal immune cells caused by co-exposure to bacterial toxins and food allergens should be considered. In our study, an OVA food allergy mouse model exposed to SEB was constructed to investigate the function of the intestinal barrier and mucosal immune status. The staining of jejunum, RNA expression in the intestine, and composition of gut flora were examined (**Figures 1**–**3**). In addition, intestinal epithelial cells, DCs, and T cells, which play an essential role in the development of food allergy, were isolated and co-stimulated with SEB and OVA *in vitro* to explore the possible cellular mechanisms of how SEB promotes the development of food allergy. The doses of SEB used in the mouse model (1 μg or 10 μg per mouse) and cell stimulation (50 ng/ml) were referenced from previous studies (23, 24). Our results provide more evidence to prove that co-exposure to SEB leads to the intestinal barrier’s damage and the activation of Th cells and DCs, thereby promoting the development of food allergy.

The OVA-sensitized mice were exposed orally to SEB to characterize the effects of SEB on the intestinal mucosa in allergic mice (**Figures 1**–**3**). Oral exposure to OVA and SEB simultaneously (OVA+Low and OVA+High groups) led to more destruction of the intestinal epithelium with distortion of the crypt and overall tissue architecture compared to the OVA group (**Figure 1B**). And, more FITC-OVA could be transformed through intestinal barrier under the co-stimulation of SEB in a Caco-2 cell monolayer model. (**Figure 1C**). More downregulated genes including *Zo-1*, *Occludin*, and *claudin-2* were found in the OVA+High group (**Figures 2A–C**) compared to the OVA group. The decreased expression of tight junction proteins and intestinal barrier disruption could lead to food allergies through abnormal exposure to allergens (25, 26). Our results suggested that co-exposure to SEB could disrupt intestinal tight junction proteins and damage the intestinal barrier, leading to increased allergen-permeation and promoting food allergy development.

In the intestine of mice, more *IL-13* and *IFN-γ* were expressed in the OVA+Low group compared to the OVA group, while more *IFN-γ* and less *IL-13*, *GATA3* was expressed in the OVA+High group compared to the OVA and OVA+Low group (**Figures 2D, E, H**). It suggests that higher Th2 polarization in the intestine could be induced under the co-exposure to low-dose SEB compared to the OVA group. In contrast, more Th1 polarization could be caused by co-exposure to high-dose SEB compared to low-dose SEB (**Figure 2D**). The activation of Th2 cells was indispensable in the development of IgE-mediated allergies, and the release of Th2 cytokines was necessary for the generation of allergens-specific IgE. And in allergic airway inflammation, exposure to SEB at the lower dose significantly boosted the secretion of allergen-specific Ig. A higher dose of SEB led to lower secretion of specific IgE in a Th2-dependent pathway (27). Our results showed that the low dose of SEB promoted the development of food allergy through the imbalance of Th1 and Th2 cells.

A co-culture of epithelial, DC, and T cells *in vitro* was designed to investigate the effect of SEB on the recognition and processing of allergens by DCs and Th cells. The co-stimulation of SEB could promote the uptake of OVA in a BMDCs-epithelial co-cultured assay (**Figure 4A**). And more expression of CD40 and CD86 could be induced under the co-stimulation with OVA and SEB compared to the OVA group (**Figures 4B, C**). The levels of costimulatory molecules, including CD28, CD40, and CD86, could also be enhanced and contribute to the activation of T cells under the stimulation of SEB (12). These activated BMDCs induced by SEB and OVA might also contribute to the activation of naïve Th cells toward Th1 and Th2 in **Figure 5**. Then, the co-stimulation with SEB and OVA could promote the differentiation of naive Th cells into Th1 and Th2 and reduce the ratio of Treg cells in the co-culture model with BMDCs (**Figure 5**). Our work provides further evidence that co-exposure to SEB could promote the transfer and uptake of OVA by DCs and then accelerate the activation of DCs or T cells through co-culture cell models. This could also be part of the evidence to reveal the potential cellular mechanism of how SEB could promote the development of food allergy. In the mice model, Th1 and Th2 cell upregulation could also be observed under exposure to OVA and SEB in our following study (The data have not yet been published.). Our present results only indicated that T cells were activated by co-stimulation of OVA and SEB, which could lead to unusual responses to OVA of T cells. Unfortunately, due to technical reasons, the purity of enriched naïve Th cells is 65.7% in alive Th cells. The procedure was followed with a naïve CD4 T cell isolation kit (Miltenyi Biotec). And we used enriched Th cells for DC-T co-culture. Indeed, the purity of the Th cells we obtained was lower than the reference values. However, the enriched CD4^+^ T cells accounted for 70% of living cells. And the expression of transcription factors was measured in the range of CD4^+^ cells. Therefore, our results could partly show changes in Th differentiation caused by the co-stimulation of SEB and OVA.

As DCs activated by SEB and OVA could uptake more allergens, promote the activation of Th cells, and play an important role in the development of food allergy (**Figures 4**, **5**), the isolated BMDCs stimulated with SEB and OVA were collected. An mRNA-seq analysis was used to gain further insights into the DCs’ response to SEB or OVA (**Figure 6F**). The C-type lectin Receptors (CLRs), as pattern-recognition receptors expressed on innate immune cells, were proven to have a detrimental role in bacterial infections, which could lead to Th2 and Th17 tendencies (28). NF-κB signaling pathway was also confirmed to play a critical role in the development of food allergy (29). Moreover, sectional fungal-derived allergens are a significant contributing factor in initiating allergic asthma through the CLRs (30). More expression of mRNAs associated with NF-KB and C-type inflammatory pathways could be found in BMDCs stimulated by OVA and SEB together compared to the OVA group (**Figure 6**). SEB could bind with MHC-II class molecules expressed on APCs with one or more variable T cell receptor (TCRs) Vb chains, through an overlapping binding region on HLA-DR (31) followed by activating protein tyrosine kinases (PTKs), LCK, and ZAP-70, leading to phospholipase Cγ (PLCγ) activation, the release of intracellular second messengers, and subsequent protein kinase C(PKC) activation. This work suggested that SEB promoted the uptake and presentation of OVA by BMDCs potentially through the activation of CLRs and NF-κB signaling pathway dependently.

Antigen-presenting and T cells have acquired a variable and nearly infinite variety of possible recognition of invading antigens during the ongoing defense against bacterial infection. At the same time, bacteria have evolved various strategies to evade recognition and clearance by immune cells. Thus, bacterial toxin pathogens can lead to different T-cell outcomes, including extensive activation, elimination, and inhibition (32). VacA expressed by *Helicobacter pylori*, YopH protein from *Yersinia*, and Lymphostatin expressed by enteropathogenic *Escherichia coli* could inhibit T cell proliferation (33–35). Heat-labile toxins expressed by *E. coli* and *Vibrio cholerae* lead to apoptosis or elimination in T cells (36). Superantigens, represented by *staphylococcal* enterotoxins, could induce a non-antigen-specific activation of T cells by cross-linking MHC and TCR with the presence of APCs, leading to rapid proliferation and release of cytokines (37, 38). Our results suggested a potential risk raised by co-exposure of enterotoxins and food allergens. The abnormal activation of DCs and Th cells induced by SEB could lead to an excessive immune response to OVA. These results may also partly explain the high association between *S. aureus* and food allergy in epidemiological investigation (2, 39). In atopic dermatitis and allergic asthma, SEB could facilitate sensitization to OVA and induce T cell proliferation, likely due to augmentation of DC migration and maturation (40, 41). These previous studies supported our inference that SEB-induced activation of DC and T cells in the intestinal mucosa may be critical evidence that SEB promotes the development of food allergy.

Additionally, the LPS was used as a common bacterial toxin leading to inflammation in various cells, including T cells (42) and DCs (43) The uptake of OVA in BMDCs and expression of CD40 and CD86 was promoted by co-stimulation of LPS (**Figure 4**). But the damage of the intestinal barrier, imbalance of Th differentiation, and dysbacteriosis in the gut was not significantly altered under co-exposure to LPS (**Figures 1**-**3**). Based on previous studies, whether LPS can promote or inhibit food allergies is still controversial (3, 42, 44), and the secretion of OVA-IgE could not be induced in the mouse model by oral administration of OVA added with LPS (Data not shown. However, the uptake and recognition of OVA in BMDCs caused by LPS still suggest a potential effect of LPS on the development of allergies.

We also want to determine whether the administration of SEB or LPS, as bacterial toxins, would lead to gut microbiome dysbiosis and then contribute to the development of food allergy. It was shown that the α-diversity of the intestinal flora was significantly decreased in the OVA allergy group. In contrast, this decrease might be partially reversed under the stimulation of SEB or LPS (**Figure 3**). In gut microbiota analysis, Beta diversity is an index to measure the similarity of microbial composition among individuals, that is, compared with Alpha diversity, it takes into account the presence and inconsistency of species among individuals. Individuals with food allergies exhibited lower diversity of the total microbiota, particularly in *Bacteroides, Parabacteroides, and Prevotella* (45, 46). Previous studies have shown that the enrichment of intestinal flora *Clostridium* and *Firmicutes* can alleviate food allergies in young infants. Increasing *Enterobacter* and *Bacteroidetes* can lead to the opposite allergic reaction (47). In our sequencing results, the intestinal microflora changes of mice in OVA food allergy groups were found mainly in the genera *Prevotella* and *Mimovotella*. *Prevotella* can promote Treg differentiation through the production of short-chain fatty acids. The produced endotoxin can also stimulate Th1 response through the TLR4 signaling pathway, which can alleviate the occurrence of food allergy (48). *Praevotella* is also considered beneficial for relieving food allergies (49). Moreover, the most significantly upregulated functional flora in the OVA allergy group was focused on translation, replication and repair, immune system, and infection disease (**Figure 3F**). These changes could be partly reversed under the co-exposure to SEB (**Figures 3G, H**). The differential functional flora of the other groups also concentrated on these aspects (**Figure S2**). It suggested that the co-exposure to SEB and OVA led to the decreased diversity of intestinal flora, the aggravated decline of intestinal flora diversity, and the worsened macroecology of intestinal flora.

## 5 Conclusion

Overall, our results suggested a potential role of SEB in exacerbating food allergy by worsening the injury of the intestinal barrier, the imbalance of mucosal Th immune, and the disequilibrium of gut flora. The abnormal activation of DCs and Th cells induced by SEB could be the potential mechanism, particularly the up-regulation of CLRs and NF-κB signaling pathways in DCs. Our findings suggest that SEB in food or environment should be considered a risk factor for allergic individuals. Accordingly, the contamination of *S. aureus* in the allergens and the potential exposure of SEB in the population need to be further evaluated for sufficient data to improve the risk management of food allergy.

## Data availability statement

The original contributions presented in the study are publicly available. This data can be found here: RNA-seq raw data is available at BIG Sub (https://ngdc.cncb.ac.cn/bioproject/) under GSA number CRA007904. 16S rRNA Gene Sequencing raw data is available at BIG Sub (https://ngdc.cncb.ac.cn/bioproject/) under GSA number CRA007879.

## Ethics statement

Ethical approval was not required for the studies on humans in accordance with the local legislation and institutional requirements because only commercially available established cell lines were used. The animal study was approved by Animal Care and Use Committees of Nanchang University. The study was conducted in accordance with the local legislation and institutional requirements.

## Author contributions

JY: Writing - original draft, Writing – review & editing, Data curation, Conceptualization, Formal analysis, Investigation, Visualization. PT: Writing – review & editing, Data curation, Conceptualization, Validation. XM: Writing – review & editing, Investigation, Resources, Visualization. YW: Writing – review & editing, Data curation, Validation. XL: Writing – review & editing, Data curation, Resources. JG: Writing – review & editing, Data curation, Validation, Funding acquisition. HC: Writing – original draft, Writing – review & editing, Data curation, Conceptualization, Project administration, Validation, Funding acquisition.
