# Supplementary material for: Oral exposure to Staphylococcus aureus enterotoxin B could promote the Ovalbumin-induced food allergy by enhancing the activation of DCs and T cells
Source: Front Immunol. 2023 Oct 16;14:1250458. doi: 10.3389/fimmu.2023.1250458 (PMC10615071; doi:10.3389/fimmu.2023.1250458)
Supplement: Supplementary file 1 [file DataSheet_1.pdf]

**Supplement Materials**

Fig.S1 The purity of the naïve Th cells and gate strategy of Th cells. (A) The purity of the naïve Th cells after isolation from spleen cells. The spleen cells and isolated naïve Th cells were stained with Fixable Viability Dye eFluor 780 and anti-murine antibodies: BV605-CD4, PerCP-CD44, and AF647-CD62L. (B) The gating strategy of Th cells. The expression of Foxp3, T-bet, and GATA3 in CD4+ cells was evaluated.

Fig. S2 The differences in functional caecal microflora between groups were performed based on KEGG. (A) The difference between the OVA group and the OVA+LPS group. (B) The difference between the OVA+Low group and the OVA+High group. (C) The difference between the OVA+Low group and the OVA+LPS group. (D) The difference between the OVA+High group and the OVA+LPS group.

Supplement Table 1 The sequences of primers.

| Name of Primers | Sequence (5' to 3') |
|---|---|
| *Gapdh*-F | CCTGTTGCTGTAGCCGTATTCA |
| *Gapdh*-R | CCAGGTTGTCTCCTGCGACTT |
| *Foxp3*-F | TGGAAAAGGAGAAGCTGGGAG |
| *Foxp3*-R | AGTACTGGTGGCTACGATGC |
| *Gata3*-F | GAGGAGGAACGCTAATGGGG |
| *Gata3*-R | CGGGTCTGGATGCCTTCTTT |
| *IFN-γ*-F | ATTGCGGGGTTGTATCTGGG |
| *IFN-γ*-R | GGAAGCACCAGGTGTCAAGT |
| *TGF-β*-F | GACTCTCCACCTGCAAGACCAT |
| *TGF-β*-R | GGGACTGGCGAGCCTTAGTT |
| *ZO1*-F | CGAGGCATCATCCCAAATAAGAAC |
| *ZO1*-R | TCCAGAAGTCTGCCCGATCAC |
| *Cldn2*-F | GGCTGTTAGGCACATCCAT |
| *Cldn2*-R | TGGCACCAACATAGGAACTC |
| *T-bet*-F | CAACAACCCCTTTGCCAAAG |
| *T-bet*-R | TCCCCCAAGCAGTTGACAGT |
| *Ocln*-F | GCTGTGATGTGTGTGAGCTG |
| *Ocln*-R | GACGGTCTACCTGGAGGAAC |

Supplement Table 2 The glossary of abbreviations

| Abbreviations | Whole name |
|---|---|
| BMDCs | Bone marrow dendritic cells |
| CLRs | C-Type Lectin Receptors |
| DCs | Dendritic cells |
| DMEM | Dulbecco's modified eagle medium |
| HE | Hematoxylin-eosin |

| | |
|---|---|
| LPS | Lipopolysaccharide |
| MHC-II | Histocompatibility Complex II |
| OVA | Ovalbumin |
| PBS | Phosphate buffered saline |
| *S. aureus* | *Staphylococcus aureus* |
| SEB | *Staphylococcus aureus* enterotoxin B |
| TCR | T cell receptor |
| Th | Helper T |
