# Supplementary figures and images for: Oral exposure to Staphylococcus aureus enterotoxin B could promote the Ovalbumin-induced food allergy by enhancing the activation of DCs and T cells
Source: Front Immunol. 2023 Oct 16;14:1250458. doi: 10.3389/fimmu.2023.1250458 (PMC10615071; doi:10.3389/fimmu.2023.1250458)

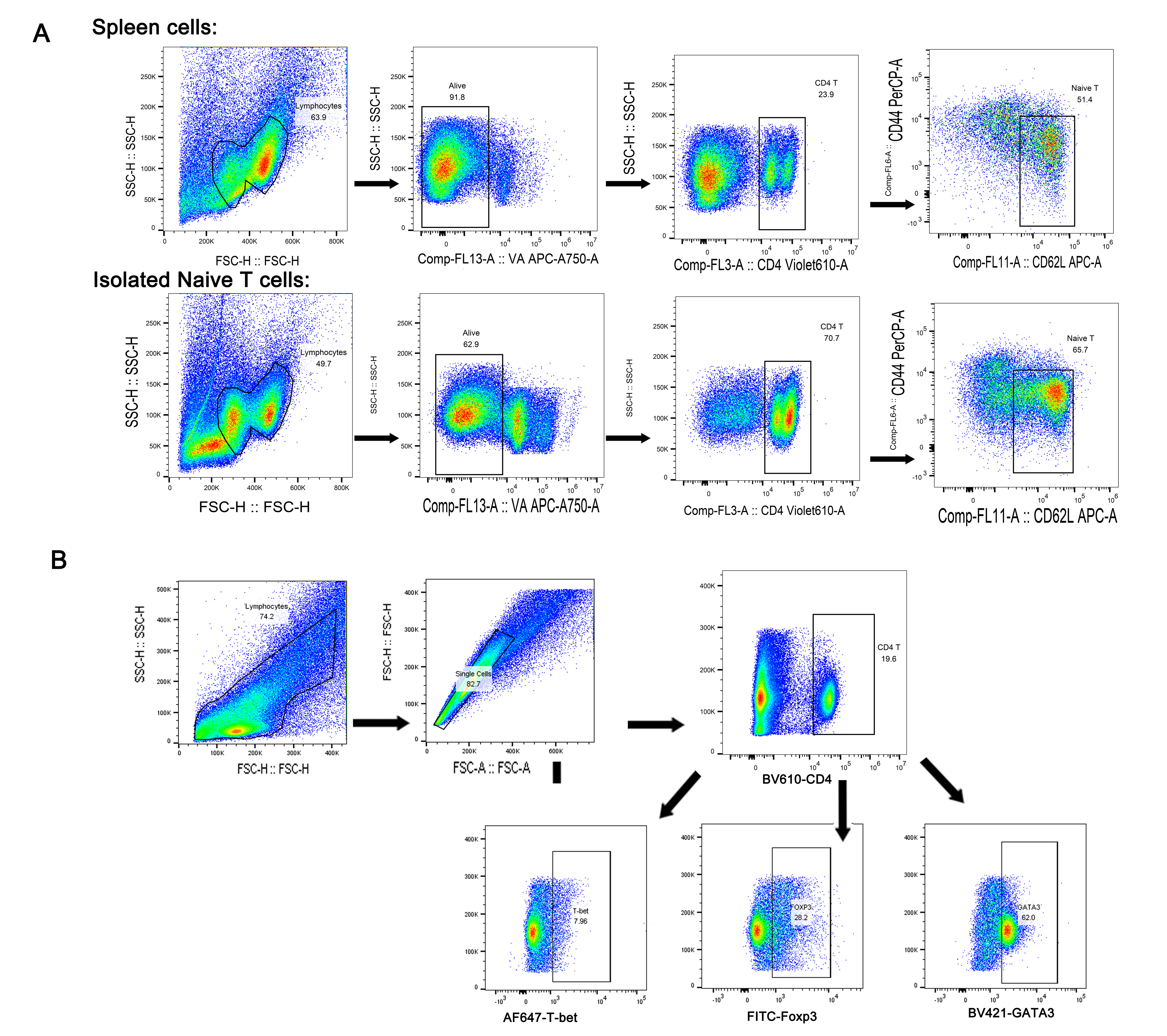

Supplement: Supplementary file 2 [file Image_1.tif]

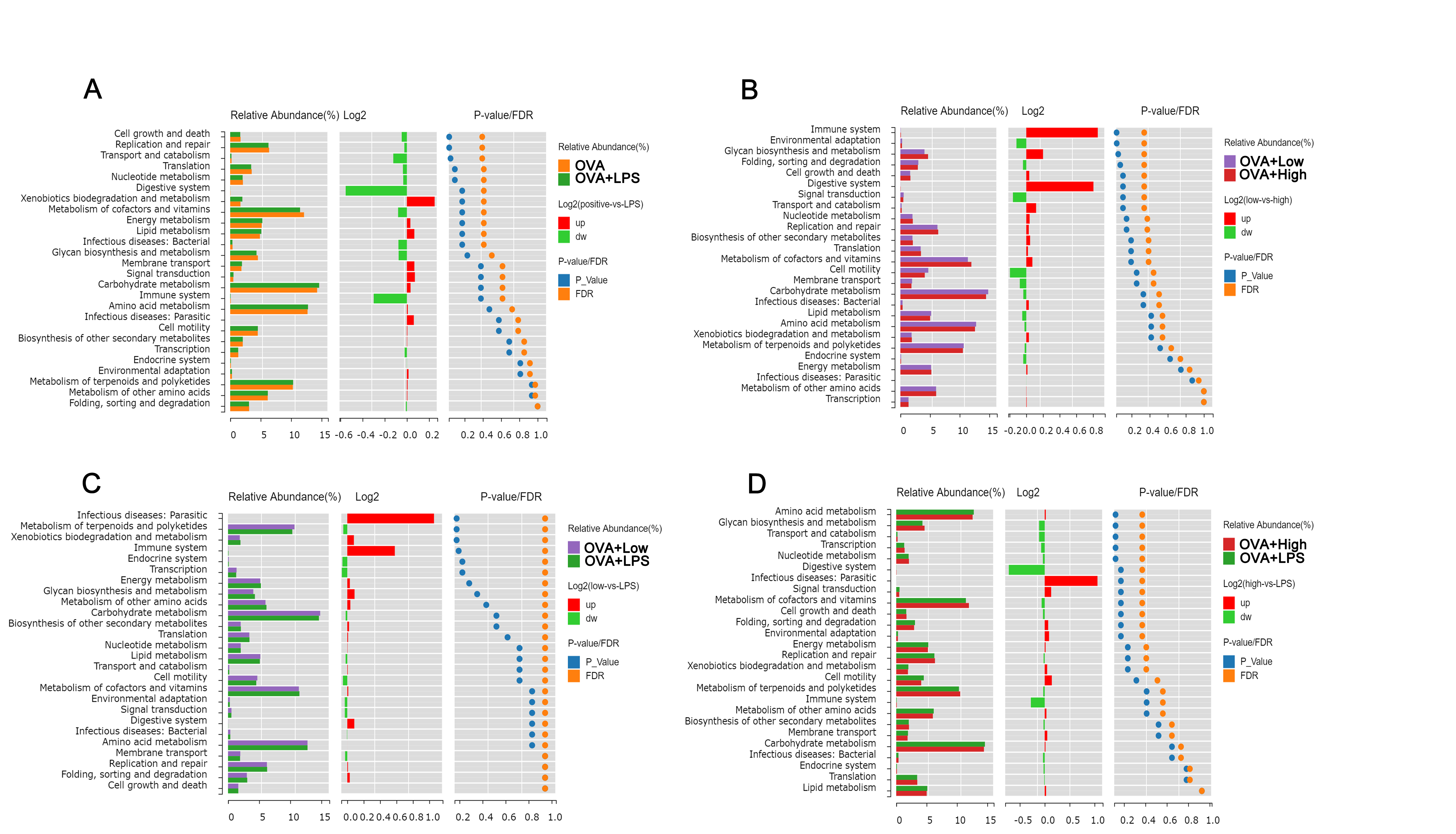

Supplement: Supplementary file 3 [file Image_2.tif]
